# Green Synthesis and Characterization of Copper Nanoparticles Using *Fortunella margarita* Leaves

**DOI:** 10.3390/polym13244364

**Published:** 2021-12-13

**Authors:** Rutaba Amjad, Bismillah Mubeen, Syed Shahbaz Ali, Syed Sarim Imam, Sultan Alshehri, Mohammed M. Ghoneim, Sami I. Alzarea, Rabia Rasool, Inam Ullah, Muhammad Shahid Nadeem, Imran Kazmi

**Affiliations:** 1 Institute of Molecular Biology and Biotechnology, The University of Lahore, Lahore 5400, Pakistan; rutabadanish23@gmail.com (R.A.); bismillah.mubeen@gmail.com (B.M.); rabia.amjad545499@gmail.com (R.R.); inamgenetics@gmail.com (I.U.); 2 School of Physical Sciences, University of the Punjab, Lahore 54590, Pakistan; shahbaz.sps@pu.edu.pk; 3 Department of Pharmaceutics, College of Pharmacy, King Saud University, Riyadh 11451, Riyadh, Saudi Arabia; simam@ksu.edu.sa (S.S.I.); salshehri1@ksu.edu.sa (S.A.); 4 Department of Pharmacy Practice, College of Pharmacy, AlMaarefa University, Ad Diriyah 13713, Riyadh, Saudi Arabia; mghoneim@mcst.edu.sa; 5 Department of Pharmacology, College of Pharmacy, Jouf University, Sakaka 72341, Aljouf, Saudi Arabia; samisz@ju.edu.sa; 6 Department of Biochemistry, Faculty of Science, King Abdulaziz University, Jeddah 21589, Makkah, Saudi Arabia

**Keywords:** characterization, CuNPs, *Fortunella* *margarita*, UV-Vis

## Abstract

The use of biomaterials in the synthesis of nanoparticles is one of the most up-to-date focuses in modern nanotechnologies and nanosciences. More and more research on green methods of producing metal oxide nanoparticles (NP) is taking place, with the goal to overcome the possible dangers of toxic chemicals for a safe and innocuous environment. In this study, we synthesized copper nanoparticles (CuNPs) using *Fortunella margarita* leaves’ extract, which reflects its novelty in the field of nanosciences. The visual observation of a color change from dark green to bluish green clearly shows the instant and spontaneous formation of CuNPs when the phytochemicals of *F. margarita* come in contact with Cu^+2^ ions. The synthesis of CuNPs was carried out at different conditions, including pH, temperature, concentration ratio and time, and were characterized with UV-Vis absorption spectra, scanning electron microscope (SEM) and X-ray diffraction (XRD). The UV-Vis analysis reveals the surface plasmon resonance property (SPR) of CuNPs, showing a characteristic absorption peak at 679 nm, while SEM reveals the spherical but agglomerated shape of CuNPs of the size within the range of 51.26–56.66 nm.

## 1. Introduction

Plants are well-known for their high dietary sources of flavonoids for humans, coronary heart disease prevention, having high free radical scavenging capacity and anticancer activity, and also exhibit anti-HIV functions, chemotaxonomic markers and antimicrobial agents [1,2,3]. They also play a key role in the maintenance of the water cycle, balancing the ecosystem, provide oxygen for maintenance of the environment, produce chemicals for drug discovery and provide wood and timber for household and furniture [4]. Nowadays, plants gain attention towards the utilization of phytochemicals in nanotechnology. Nanotechnology is an advanced discipline in which particles are studied in the range of 10^−7^ to 10^−9^ m [5]. Nanotechnology has a vast applicability in areas such as environmental sciences, bio-nanotechnology, applied microbiology, medicine and drug–gene delivery systems, quantum dots, surface-enhanced Raman scattering (SERS), chemistry, space and chemical industry, energy science, mechanics, electronics, optics and optoelectronic devices [6,7,8,9,10,11,12,13,14,15]. 

**Figure 1:**
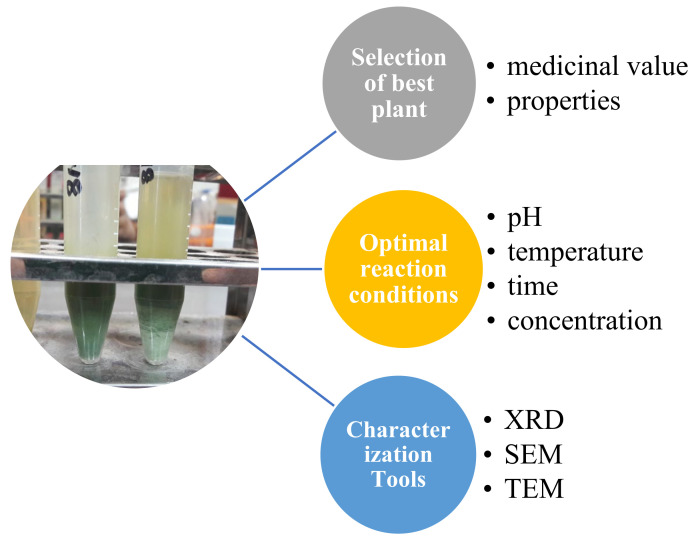
Important aspects for the production of green nanoparticles.

Among them, bio-nanotechnology (green synthesis) is an eco-friendly and cost-effective method for the formation of nanoparticles by using simple prokaryotic bacterial cells for complex eukaryotic plants, because they do not contain any usage or production of toxic chemicals and can easily cope with higher production because they do not require energy, temperature and pressure. Whereas chemical and physical methods of nanoparticle synthesis may adsorb toxic chemicals on the surface that may lead to highly adverse reactions in the medical field [15,16,17,18,19,20]. Green synthesis also acquires some important aspects for producing stable and well-characterized nanoparticles, such as selection of best organism, optimal conditions for reaction and characterization tools (Figure 1). To select the best plant for green synthesis, one should know about its detoxification and potential in heavy metal accumulation, while reaction conditions should also be known, such as pH, temperature, etc. [10].

Various plants have been utilized for the production of nanoparticles due to their medicinal values and properties. *Aleo vera*, *Asparagus adscendens*, *Allium sativum*, *Dodonaea viscosa*, *Citrus medica*, *Punica granatum*, *Eclipta prostrata, Dioscorea bulbifera, Iris pseudacorus, Calotropis procera, Citrus limon, Leucas chinensis, Euphorbia esula, Punica gratum, Ocimum sanctum, Lawsonia inermis, Magnolia kobus, Citrus limon, Aelge marmelos, Syzygium aromaticum, Alchornea laxiflora, Camellia sinensis, Zingiber officinale* and *Allium sativum, Phyllanthus embilica, Eucalyptus* and *Artabotrys odoratissimus* have been previously used for the synthesis of copper nanoparticles (CuNPs) [21,22,23,24,25,26,27,28,29,30,31,32,33,34,35,36,37,38,39,40,41,42,43,44,45,46]. *Fortunella margarita*, a close relative of Citrus, is a fruitful, small, bushy perennial tree belonging to the Rutaceae family [47]. For the present study, *Fortunella margarita* is used in the production of copper nanoparticles. Nowadays, CuNPs are taking the place of gold and silver nanoparticles because they are a promising contender for the future, but CuNPs are highly oxidant, have a high melting point and electrical conductivity, low electrochemical migration behavior, small size, shape and oxidation resistance, high surface/volume ratio and low cost [48,49,50,51,52,53].

**Figure 2:**
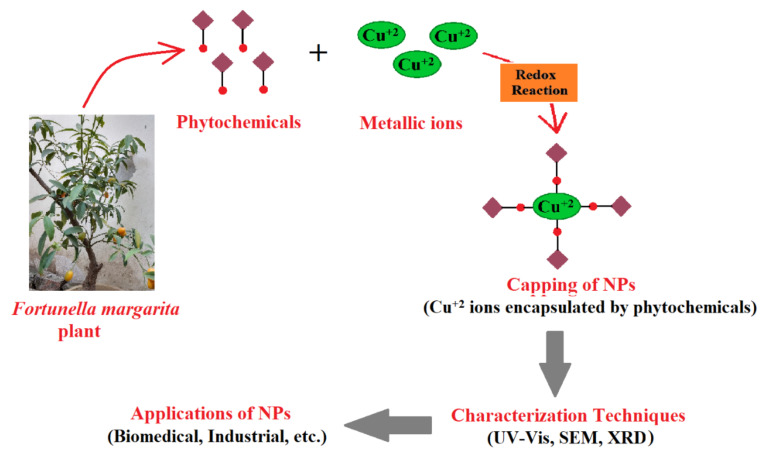
A diagrammatic sketch representing the mechanism and utilization of synthesized nanoparticles using plant extracts.

The mechanism of in vitro nanoparticle synthesis from plant extracts (Figure 2) involves three main phases: (1) nucleation: which is the activation phase of protons of various functional groups to reduce metallic ions, i.e., cations are super-saturated to form hydroxyl complexes for the formation of reactive oxygen species (ROS), (2) Ostwald ripening or aggregation or coarsening: the growth phase in which nanoparticles aggregate into larger particles (i.e., aggregation of nanoparticles (NPs) by conversion of high-energy state to low-energy conformations) by forming different irregular shapes, such as nanoprisms, nanotubes, nanohexahedrons, nanorods, etc., and (3) bio-reduction: the termination phase, which determines the final shape of nanoparticles, and acquires the favorable energetic conformation to stabilize metallic nanoparticles. The higher the amount of reducing agents, the higher the production of small-sized nanoparticles should be promoted [54,55,56]. Plant biomolecules such as flavonols, acids, enzymes, polysaccharides, etc., are complex compounds, yet environmentally benign after bio-reduction of metal nanoparticles, i.e., secondary metabolites (phenolic compounds) have the ability to bind or conjugate with metal ions to purify compounds and are used in drug discovery [10,57].

The morphology and size of nanoparticles depend on various physical measurements, such as light, temperature, pH, substrate–metal concentration, nutrients and enzymes [16,17,18,19,20]. Optimization of light and temperature directly affect the size, morphology and the rate of the reaction. These factors may facilitate the researchers to overcome the limitations of the synthesis of nanoparticles. The morphological diversity of nanostructures depends on the concentration, composition and contribution of the metallic ions to produce different shapes, such as triangles, spheres, cubes, pentagons, hexagons, ellipsoids, nanorods, nanowires, nanoprisms, nanotubes, nanohexahedrons, nanoflowers and nanobuds. Nanoparticles reveal new and improved properties depending upon their surface area, morphology, size and distribution of the particles [6,7,8,9,10,11,12,13,14,15]. Green nanoparticles (GNPs) can be characterized by various techniques, such as X-ray diffraction (XRD), Fourier transform infrared spectrum analysis (FT-IR), UV-visible absorption spectrum, scanning electron microscope (SEM), transmission electron microscope (TEM) and atomic force microscopy (AFM). 

The present study is based on bio-nanotechnology, with the emphasis on green synthesis and characterization of copper nanoparticles by using *Fortunella margarita* leaves.

## 2. Materials and Methods

All chemicals used were of analytical grade, such as copper sulphate, ethanol, etc. The work area and apparatuses used were sterile, such as the falcon tubes, beakers, graduated measuring cylinder, funnel, Eppendorf tubes, pH meter, lyophilizer (VaCO_2_ lyophilizer, Zhejiang Guanfeng Food Machinery Co., Ltd., Shaoxing, China), centrifuge (HITACHI 1 High-Speed Refrigerated Centrifuge CR22N, Tokyo, Japan), UV-spectrophotometer and many others.

### 2.1. Synthesis of Copper Nanoparticles

Five grams of leaves of *Fortunella margarita* (kumquat) were washed and crushed finely into a thin paste by using pestle mortar. The paste was diluted with distilled water up to 100 mL and was kept at room temperature. After 1 h, the mixture was filtered to obtain the phytochemicals (reducing agents) for the reaction. The filtrate was then mixed with 1 mM of copper sulphate (CuSO_4_·5H_2_O) (Riedel-de-Haen Ag Seelze Havvonee^TM^, Seelze, Germany) solution and was kept in a water bath at 70 °C for 30 min. Synthesized CuNPs were then collected and washed thrice by using a centrifuge (HITACHI 1 High-Speed Refrigerated Centrifuge CR22N) at 12,000 rpm for 1 h at 30 °C. After washing, copper nanoparticles were lyophilized/dried (VaCO_2_ lyophilizer) to obtain the powdered copper nanoparticles. 

### 2.2. Effect of Different Parameters on the Production of Copper Nanoparticles

Leaf extract of *Fortunella margarita* (Kumquat) and copper sulphate solution was mixed in the ratios of 1:2; 1:3 and 1:4 to find the best yielding points with concentration ratio, pH and temperature for the better production of copper nanoparticles (Table 1 Table 2 and Table 3). 

### 2.3. Characterization of CuNPs

For UV-Vis, aliquots of CuNPs were prepared by dissolving 5 mg of CuNPs in 5 mL of sterile distilled water. They were then vortexed and sonicated alternately for better segregation of each nanoparticle. Afterwards, 2 mL of CuNPs was loaded in a Quartz Cuvette and absorbance was measured through the UV-Vis spectrophotometer (CECIL-7400S, Cecil Instruments Services Ltd., London, UK) in the range of 400–800 nm. The SEM analysis of dried powder of CuNPs was used for the detection of size and shape of the nanoparticles. The analysis was performed with a FEI Nova Nano SEM 450 and the photomicrographs were taken with specific magnifying lenses. The XRD pattern can be analyzed by spreading a thin layer of well-grinded dried CuNPs all along the glass slide, which was inserted into the XRD chamber. The pattern of X-ray diffraction (XRD) of synthesized CuNPs was recorded on an X-ray diffraction meter (Philips PANalytical X′pert Powder, Malvern Panalytical Ltd., Malvern, UK), with a scan range of 0–110, step size of 0.02 and time per step of 20–30 s.

## 3. Results

### 3.1. Synthesis of CuNPs

**Figure 3:**
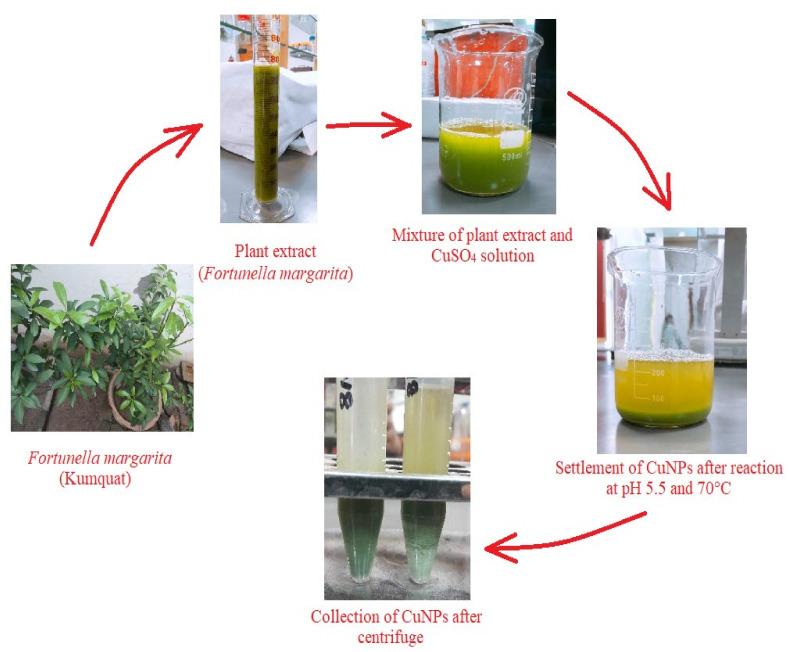
A schematic representation of formation, settlement and collection of CuNPs obtained from *Fortunella margarita* leaves’ extract.

When extract of *Fortunella margarita* (kumquat) was mixed with 1 mM of CuSO_4_ solution, CuNPs formed instantly and can be visibly seen with the naked eye, observing the opacity and color change. The color of the extract was changed from dark green to bluish green and the cloudy appearance of the mixture turned into a clear yellow green solution with settled nanoparticles. The synthesized nanoparticles were then centrifuged thrice for their collection, purification and were lyophilized to obtain powdered CuNPs. This visual observation of color change explains the formation of small-sized nanoparticles (Figure 3). 

### 3.2. Effect of Different Parameters on the Production of CuNPs

The concentration ratio of *F. margarita* leaves’ extract to CuSO_4_ solution, time period, pH of CuSO_4_ solution and temperature of the reaction were optimized for excess and surplus production of nanoparticles. All of these variables varied differently, which resulted in different amounts of synthesized CuNPs. The color change was visually observed. High productivity of CuNPs, i.e., 5 mL, was visibly observed, with a bluish green color at a concentration ratio of 1:2, pH 5.5 and temperature 70 °C in 30 min. By increasing the concentration of the CuSO_4_ solution, the production of CuNPs decrease because less bioactive compounds (phytochemicals) are present, which react with Cu^+2^ ions, while doubling the amount of CuSO_4_ immediately reacts with Cu^+2^ ions and forms small-sized nanoparticles which can be visually seen by their settlement (Table 1). 

The effect of pH plays an important role in the production of nanoparticles. The acidic medium (pH 5.5) maximizes the CuNPs production at a faster rate than in basic medium (pH 7.5). This effect of pH on CuNPs production is well-explained by temperature (70 °C), which lessens the events of nucleation at pH 7.5, which causes a reduction in availability of Cu^+2^ ions to phytochemicals, resulting in the agglomeration of CuNPs (Table 2). 

Variable temperature is another important optimization tool in the production of NPs with different morphological states and sizes. As the temperature rises, more nucleation events take place, which helps in the formation of instant and small-sized CuNPs, which was visually observed (Table 3). 

### 3.3. UV-Vis Spectrophotometry

**Figure 4:**
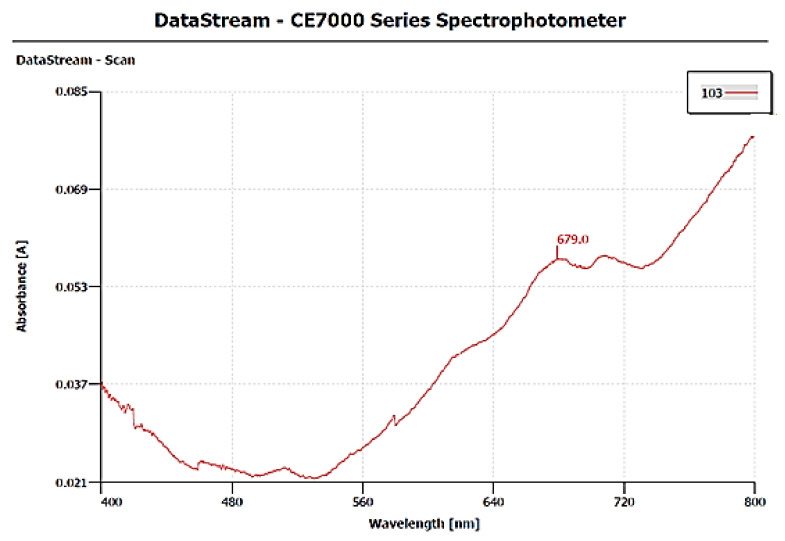
UV-Vis spectrum shows absorption peak of synthesized CuNPs.

The surface plasmon resonance property (SPR) of CuNPs was monitored by a UV-visible spectrophotometer (DataStream-CE7000 Series Spectrophotometer) within the range of 400–800 nm. UV-Vis spectral analysis of CuNPs shows a characteristic absorption peak at 679 nm, which exhibits a SPR property within 9 min. The red-colored bands indicate the presence of metallic copper, hence providing evidence for the formation of CuNPs (Figure 4). 

### 3.4. SEM Analysis

**Figure 5:**
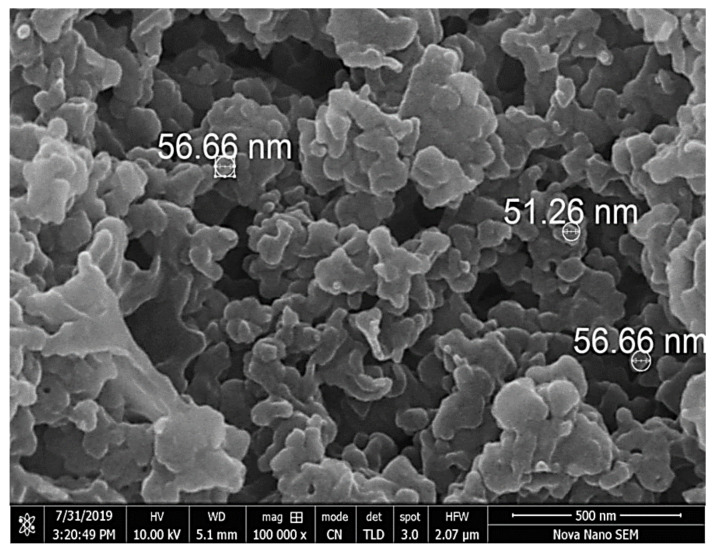
Photomicrograph of SEM analysis of synthesized CuNPs at 100,00x magnification scale in the size range of 500 nm.

The morphology of the synthesized CuNPs was studied under the FEI Nova Nano SEM 450 in the size range of 500 nm. Nanoclusters show the agglomeration of CuNPs, but the high magnification power (100,000×) exhibits an average diameter of about 51.26 to 56.66 nm, and CuNPs are spherical in shape. (Figure 5).

### 3.5. XRD

**Figure 6:**
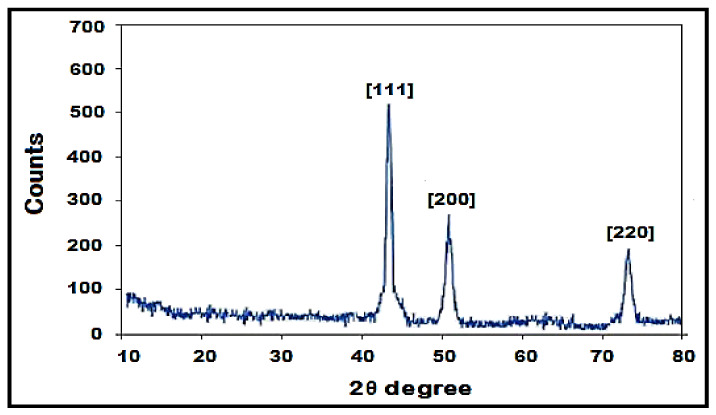
XRD pattern of synthesized Copper nanoparticles using *Fortunella margarita* leaves’ extract.

The X-ray diffraction analysis shows the crystalline structure of copper nanoparticles with prominent peaks. Bragg’s reflection of copper nanoparticles shows diffraction peaks around 2θ = 43.4°, 50.3° and 74.39°, representing [111], [200] and [220] crystallographic planes of face-centered cubic (fcc). (Figure 6).

## 4. Discussion

Nanotechnology, a vast arena, is gaining much attention, and especially bio-nanotechnology (micro-organisms to higher plants) is emphasized globally nowadays as chemical synthesis affords low production of nanoparticles and is toxic and non-eco-friendly. The present study deals with the formation, optimization and characterization of the synthesized CuNPs produced from *Fortunella margarita* (kumquat) leaves’ extract. The study was performed under a controlled environment and supervision. The visual observation in the formation of CuNPs was the color change, i.e., from green to bluish green, and the settlement of copper nanoparticles with yellow green supernatant confirms the full bio-reduction of phytochemicals present in the *Fortunella margarita* leaves (Figure 3). The main advantage of using the green route for the production of CuNPs is stabilization [58]. Capping agents present in phytochemicals help CuNPs to stabilize for more than 30 days, while chemical production of CuNPs makes them oxidize and settle down after 24 h, along with large-size CuNPs production [38]. 

The synthesis of copper nanoparticles is a difficult task for a researcher to find the best valuable points, such as pH, temperature, concentration ratio and time. Time plays an important role in the synthesis of nanoparticles. In some cases, a color change occurs within 30 min, while sometimes it takes up to 48 h. This color change gives the visual observation of the production of CuNPs, which was confirmed with UV-Vis studies. The CuNPs obtained from *Aloe vera* flower extract changed color from light green to dark green in 30 min, and the formation of nanoparticles was confirmed with UV-Vis studies with the SPR at 578 nm [22]. CuNPs synthesized from *Asparagus adscendens* leaves’ extract showed a color change from brown to sea green in 1 h, which was monitored via UV-Vis spectroscopy with an absorption peak within the range of 500–700 nm [23]. *Allium sativum* synthesized CuNPs showed a gradual change in color from straw yellow to light green when both solutions were mixed together, and finally to a bright light-green solution after 48 h of reaction at room temperature, while the CuNPs were monitored for their presence at 580 nm via the UV-Vis spectrophotometer [24]. The concentration of phytochemicals present in the plant extract plays key role in the formation and stabilization of CuNPs. By increasing the plant extract concentration, the reduction of Cu^+2^ ions will be faster, resulting in the decreased size of CuNPs [25]. In addition to phytochemical concentration, the type of copper salt and its concentration also affects the morphology, size and productivity of CuNPs. Copper chloride forms triangular or tetrahedron nanoparticles, copper acetate produces rod-shaped nanoparticles and copper sulphate helps in the formation of spherical nanoparticles, while the increased copper salt concentration causes an increase in the size of CuNPs [59]. The second important parameter for the production and size estimation of CuNPs is pH. Higher pH produces small-sized nanoparticles as compared to lower pH values. The difference is due to the reduction rate of Cu^+2^ ions with the phytochemicals. By adding copper chloride (CuCl_2_) solution in *Dodonaea viscosa* extract, no CuNPs were formed. Instead, the pH of the reaction mixture was changed to basic medium for the formation of CuNPs [25]. This relation between pH and copper salt was also reported, where lower pH forms large-size nanoparticles (rod-shaped or triangular) while higher pH produces small-sized nanoparticles (spheres) [60]. At pH 10, CuCl_2_ forms pure CuNPs with hydrazine in aqueous cetyltrimethyl ammonium bromide (CTAB) solution, while at pH 8, impurities such as copper oxide nanoparticles (CuO-NPs) were formed [61]. In addition, the chemical reduction method for the synthesis of CuNPs also proves the same phenomenon; as the pH increases (6 to10), particle size decreases from 18 to 9 nm. However, when the pH exceeds 11, the particle size increases [62].

The third parameter to raise the synthesis rate of CuNPs is temperature. By increasing the temperature, the availability of copper ions to the phytochemicals also increases and reduces the risks of secondary processes. The same trend was observed with silver and gold nanoparticles synthesized via different plant extracts [38]. The synthesized CuNPs via the green route showed the effect of temperature on production, whereby low temperature will cease production to half, while higher temperatures enable the Cu^+2^ ions to reduce much faster to form small nanoparticles, but aggregates were also formed. Secondary metabolites are the main reason for the reduction of metal salt (copper, silver, etc.) into nanoparticles, and they provide prevention against aggregation of NPs [34]. Aggregation of nanoparticles can be caused by low pH (acidic medium), i.e., pH 2, which causes a reduction in nucleation events which ultimately leads to agglomeration [63]. Along with pH, particle size also plays an important role in agglomeration. Agglomeration is a phenomenon in which nanoparticles lower the surface energy, resulting in a decrease in surface area by increasing the particle size in a liquid phase. Therefore, agglomeration of NPs increases due to a decrease in the particle size. Cerium oxide nanoparticles (CeO_2_-NPs) cause agglomeration by minimizing the interface energy [64]. 

For characterization of CuNPs, many techniques were used. Less separated CuNPs can be visualized at shorter wavelengths with broadened peaks of SPR [45]. The present study depicted the formation of CuNPs with an absorption peak of 679 nm, which shows well-stabilized particles with the diameter of about 51–56 nm (Figure 4). Well-dispersed and stable CuNPs were characterized at 659 nm by the UV-Vis spectrum, and the formation was supported by SEM images with the diameter of 67–99 nm [43]. The average-sized CuNPs of 20 nm had an absorption peak of 631 nm when synthesized by Citron juice (*Citrus medica* Linn) extract [26]. The biogenic synthesis of CuNPs with *Asparagus adscendens* showed that SPR ranged between 500 and 700 nm, with an average size of 40–100 nm CuNPs [34]. A small aliquot of CuNPs was used for the detection of SPR (Surface Plasmon Resonance Property). CuNPs can also form nanoclusters ranging from 150 to 200 nm, but high magnification of SEM revealed that the spherical CuNPs ranged from 40 to 45 nm [41]. CuNPs formed from the *Punica granatum* seeds extract were found to be in the size range of 40–80 nm, which proves that CuNPs have different size ranges according to different contributing factors [27]. Besides, biological synthesis of CuNPs from *Pseudomonas stutzeri* indicated the average size of CuNPs to be in the range of 50–150 nm [65]. The study proposed the diameter of CuNPs to be in the range of 51.26–56.66 nm. The size was confirmed in the magnification range of 500 nm (Figure 5). XRD was used to evaluate the peak intensity, position and width of the nanoparticles, which confirms the purity and formation of nanoparticles [66]. The peaks in Figure 6 confirmed the formation of CuNPs and showed them to be pure from impurities, and this can be proven by matching the information provided by the Joint Committee of Powder Diffraction Standards (JCPDS) (File No. 089-2838).

## 5. Conclusions

Extracellular biogenic synthesis of CuNPs from *Fortunella margarita* leaves’ extract is the novelty of this study, as this plant has not been used before for the synthesis of CuNPs. The approach used for the synthesis was eco-friendly, non-toxic, rapid and cheap. The characterization tools explain the stability of CuNPs for future use. This study provided an opportunity to synthesize CuNPs via natural products, which could be beneficial to apply in various techniques, such as drug formulation, drug-delivery systems, biomedical applications, etc., in the future.

## Figures and Tables

**Table 1:** Production of CuNPs at constant pH and temperature with variable concentration ratios.

Concentration Ratio	pH (Cu)	Temperature T(°C)	Time Period	Production of NPs (mL)
1:4	5.5	30	2 days	0.4
1:3	5.5	30	3 h	1
1:2	5.5	30	2 h	2

**Table 2:** Production of CuNPs at constant concentration ratio and temperature with variable pH.

Concentration Ratio	pH (Cu)	Temperature (°C)	Time Period	Production of NPs (mL)
1:2	5	70	1 day	1.5
1:2	5.5	70	30 min	5
1:2	6	70	2 h	1
1:2	7	70	2 h	1
1:2	7.5	70	3 h	0.75

**Table 3:** Production of CuNPs at constant pH and concentration ratio with variable temperature.

Concentration Ratio	pH (Cu)	Temperature (°C)	Time Period	Production of NPs (mL)
1:2	5.5	30	2 h	2
1:2	5.5	50	1 h	2.5
1:2	5.5	70	30 min	5
1:2	5.5	90	2 days	3

## Data Availability

All required data is available with the first author.

## References

[1] Yao L.H., Jiang Y.M., Shi J., Tomas-Barberan F.A., Datta N., Singanusong R., Chen S.S. (2004). Flavonoids in food and their health benefits. Plant Food Hum. Nutr..

[2] Cushnie T.T., Lamb A.J. (2005). Antimicrobial activity of flavonoids. Int. J. Antimicrob. Agents..

[3] Robards K., Antolovich M. (1995). Methods for assessing the authenticity of orange juice. A Review. Analyst.

[4] Seth M.K. (2003). Trees and their economic importance. Bot. Rev..

[5] Martin C.R. (2006). Welcome to nanomedicine. Nanomedicine.

[6] Nalwa H.S. (2002). Handbook of Thin Film Materials.

[7] Sharma V.K., Yngard R.A., Lin Y. (2009). Silver nanoparticles: Green synthesis and their antimicrobial activities. Adv. Colloid Interface Sci..

[8] Jahn W. (1999). Chemical aspects of the use of gold clusters in structural biology. J. Struct. Biol..

[9] Murphy C. (2008). Sustainability as an emerging design criterion in nanoparticle synthesis and applications. J. Mater. Chem..

[10] Iravani S. (2011). Green synthesis of metal nanoparticles using plants. Green Chem..

[11] Senapati S. (2005). Biosynthesis and Immobilization of Nanoparticles and Their Applications. Ph.D. Thesis.

[12] Klefenz H. (2004). Nanobiotechnology: From molecules to systems. Eng. Life Sci..

[13] Goodsell D.S. (2004). Bionanotechnology: Lessons from Nature.

[14] Tian Z.Q., Ren B. (2004). Adsorption and reaction at electrochemical interfaces as probed by surface-enhanced Raman spectroscopy. Annu. Rev. Phys. Chem..

[15] Song J.Y., Kim B.S. (2009). Rapid biological synthesis of silver nanoparticles using plant leaf extracts. Bioprocess. Biosyst. Eng..

[16] Ahmad A., Mukherjee P., Senapati S., Mandal D., Khan M.I., Kumar R., Sastry M. (2003). Extracellular biosynthesis of silver nanoparticles using the fungus *Fusarium oxysporum*. Colloids Surf. B..

[17] Shankar S.S., Rai A., Ankamwar B., Singh A., Ahmad A., Sastry M. (2004). Biological synthesis of triangular gold nanoprisms. Nat. Mater..

[18] Ankamwar B., Damle C., Ahmad A., Sastry M. (2005). Biosynthesis of gold and silver nanoparticles using *Emblica officinalis* fruit extract, their phase transfer and transmetallation in an organic solution. J. Nanosci. Nanotechnol..

[19] Huang J., Li Q., Sun D., Lu Y., Su Y., Yang X., Wang H., Wang Y., Shao W., Ning H. (2007). Biosynthesis of silver and gold nanoparticles by novel sundried *Cinnamomum camphora* leaf. Nanotechnology.

[20] Korbekandi H., Iravani S., Abbasi S. (2009). Production of nanoparticles using organisms. Crit. Rev. Biotechnol..

[21] Karimi J., Mohsenzadeh S. (2015). Copper Nanoparticles Using Flower Extract of *Aloe Vera*. Synth. React. Inorg. Met. Org. Nano Met. Chem..

[22] Thakur S., Sharma S., Thakur S., Rai R. (2018). Green synthesis of copper nano-particles using *Asparagus adscendens roxb*. Root and leaf extract and their antimicrobial activities. Int. J. Curr. Microbiol. Appl. Sci..

[23] Joseph A.T., Prakash P., Narvi S.S. (2016). Phytofabrication And Characterization of Copper Nanoparticles Using *Allium sativum* And Its Antibacterial Activity. Int. J. Sci. Eng. Techn..

[24] Daniel S.K., Vinothini G., Subramanian N., Nehru K., Sivakumar M. (2013). Biosynthesis of Cu, ZVI, and Ag nanoparticles using *Dodonaea viscosa* extract for antibacterial activity against human pathogens. J. Nanopart. Res..

[25] Shende S., Ingle A.P., Gade A., Rai M. (2015). Green synthesis of copper nanoparticles by *Citrus medica* Linn. (Idilimbu) juice and its antimicrobial activity. World, J. Microbiol. Biotechnol..

[26] Nazar N., Bibi I., Kamal S., Iqbal M., Nouren S., Jilani K., Umair M., Ata S. (2018). Cu nanoparticles synthesis using biological molecule of *P. granatum* seeds extract as reducing and capping agent: Growth mechanism and photo-catalytic activity. Int. J. Biol. Macromol..

[27] Chung I.M., Abdul Rahuman A., Marimuthu S., Vishnu Kirthi A., Anbarasan K., Padmini P., Rajakumar G. (2017). Green synthesis of copper nanoparticles using *Eclipta prostrata* leaves extract and their antioxidant and cytotoxic activities. Exp. Ther. Med..

[28] Manceau A., Nagy K.L., Marcus M.A., Lanson M., Geoffroy N., Jacquet T., Kirpichtchikova T. (2008). Formation of metallic copper nanoparticles at the soil− root interface. Environ. Sci. Technol..

[29] Ghosh S., More P., Nitnavare R., Jagtap S., Chippalkatti R., Derle A., Kitture R., Asok A., Kale S., Singh S. (2015). Antidiabetic and antioxidant properties of copper nanoparticles synthesized by medicinal plant *Dioscorea bulbifera*. J. Nanomed. Nanotechnol..

[30] Harne S., Sharma A., Dhaygude M., Joglekar S., Kodam K., Hudlikar M. (2012). Novel route for rapid biosynthesis of copper nanoparticles using aqueous extract of *Calotropis procera* L. latex and their cytotoxicity on tumor cells. Colloids Surf. B..

[31] Amer M.W., Awwad A.M. (2021). Green synthesis of copper nanoparticles by *Citrus limon* fruits extract, characterization and antibacterial activity. Chem Int..

[32] Hase J., Bharati G., Deshmukh K., Phatangre K., Rahane N., Dokhe Shital A. (2016). Synthesis and characterization of Cu nanoparticles of *Leucas chinensis* L plant. Eur. J. Pharm. Med Res..

[33] Nasrollahzadeh M., Sajadi S.M., Khalaj M. (2014). Green synthesis of copper nanoparticles using aqueous extract of the leaves of *Euphorbia esula* L and their catalytic activity for ligand-free Ullmann-coupling reaction and reduction of 4-nitrophenol. RSC Adv..

[34] Kaur P., Thakur R., Chaudhury A. (2016). Biogenesis of copper nanoparticles using peel extract of *Punica granatum* and their antimicrobial activity against opportunistic pathogens32. Green Chem. Lett. Rev..

[35] Shende S., Gaikwad N., Bansod S. (2016). Synthesis and evaluation of antimicrobial potential of copper nanoparticle against agriculturally important phytopathogens. Synthesis.

[36] Cheirmadurai K., Biswas S., Murali R., Thanikaivelan P. (2014). Green synthesis of copper nanoparticles and conducting nanobiocomposites using plant and animal sources. RSC Adv..

[37] Lee H.J., Lee G., Jang N.R., Yun J.H., Song J.Y., Kim B.S. (2011). Biological synthesis of copper nanoparticles using plant extract. Nanotechnology.

[38] Jayandran M., Haneefa M.M., Balasubramanian V. (2015). Green synthesis of copper nanoparticles using natural reducer and stabilizer and an evaluation of antimicrobial activity. J. Chem. Pharm. Res..

[39] Kulkarni V., Kulkarni P. (2014). Synthesis of copper nanoparticles with *Aegle marmelos* leaf extract. Nanosci. Nanotechnol..

[40] Subhankari I., Nayak P.L. (2013). Synthesis of copper nanoparticles using *Syzygium aromaticum* (Cloves) aqueous extract by using green chemistry. World J. Nano. Sci. Technol..

[41] Olajire A.A., Ifediora N.F., Bello M.D., Benson N.U. (2018). Green synthesis of copper nanoparticles using *Alchornea laxiflora* leaf extract and their catalytic application for oxidative desulphurization of model oil. Iran J. Sci. Technol. Trans. A Sci..

[42] Kiranmai M., Kadimcharla K., Keesara N.R., Fatima S.N., Bommena P., Batchu U.R. (2017). Green synthesis of stable copper nanoparticles and synergistic activity with antibiotics. Indian J. Pharm. Sci..

[43] El-Refai A., Ghoniem A., El-Khateeb A.Y., Hassaan M.M. (2018). Eco-friendly synthesis of metal nanoparticles using ginger and garlic extracts as biocompatible novel antioxidant and antimicrobial agents. J. Nanostructure Chem..

[44] Caroling G., Vinodhini E., Ranjitham A.M., Shanthi P. (2015). Biosynthesis of copper nanoparticles using aqueous *Phyllanthus embilica* (Gooseberry) extract-characterization and study of antimicrobial effects. Int. J. Nano Chem..

[45] Kolekar R., Bhade S., Kumar R., Reddy P., Singh R., Pradeepkumar K. (2015). Biosynthesis of copper nanoparticles using aqueous extract of *Eucalyptus sp*. plant leaves. Curr. Sci..

[46] Kathad U., Gajera H.P. (2014). Synthesis of copper nanoparticles by two different methods and size comparison. Int. J. Pharm. Bio. Sci..

[47] Kumamoto H., Matsubara Y., Iizuka Y., Okamoto K., Yokoi K. (1985). Structure and hypotensive effect of flavonoid glycosides in kinkan (*Fortunella japonica*) peelings. Agr. Biol. Chem..

[48] Rafique M., Sadaf I., Rafique M.S., Tahir M.B. (2017). A review on green synthesis of silver nanoparticles and their applications. Artif. Cells Nanomed. Biotechnol..

[49] Crooks R.M., Zhao M., Sun L., Chechik V., Yeung L.K. (2001). Dendrimer-encapsulated metal nanoparticles: Synthesis, characterization, and applications to catalysis. Acc. Chem. Res..

[50] Hasan S., Singh S., Parikh R.Y., Dharne M.S., Patole M.S., Prasad B.L.V., Shouche Y.S. (2008). Bacterial synthesis of copper/copper oxide nanoparticles. J. Nanosci. Nanotechnol..

[51] Parikh R.Y., Singh S., Prasad B.L.V., Patole M.S., Sastry M., Shouche Y.S. (2008). Extracellular synthesis of crystalline silver nanoparticles and molecular evidence of silver resistance from *Morganella*
*sp.: Towards* understanding biochemical synthesis mechanism. ChemBioChems.

[52] Magdassi S., Grouchko M., Kamyshny A. (2010). Copper nanoparticles for printed electronics: Routes towards achieving oxidation stability. Materials.

[53] Yang J.G., Zhou Y.L., Okamoto T., Bessho T., Satake S., Ichino R., Okido M. (2006). Preparation of oleic acid-capped copper nanoparticles. Chem. Lett..

[54] Makarov V.V., Love A.J., Sinitsyna O.V., Makarova S.S., Yaminsky I.V., Taliansky M.E., Kalinina N.O. (2014). “Green” nanotechnologies: Synthesis of metal nanoparticles using plants. Acta Nat..

[55] Si S., Mandal T.K. (2007). Tryptophan-based peptides to synthesize gold and silver nanoparticles: A mechanistic and kinetic study. Chem. Eur. J..

[56] Kim J., Rheem Y., Yoo B., Chong Y., Bozhilov K.N., Kim D., Sadowsky M.J., Hur H.G., Myung N.V. (2010). Peptide-mediated shape-and size-tunable synthesis of gold nanostructures. Acta Biomater..

[57] Marslin G., Sheeba C.J., Franklin G. (2017). Nanoparticles alter secondary metabolism in plants via ROS burst. Front. Plant Sci..

[58] Varshney R., Bhadauria S., Gaur M.S., Pasricha R. (2010). Characterization of copper nanoparticles synthesized by a novel microbiological method. J. Mater..

[59] Shankar S., Rhim J.W. (2014). Effect of copper salts and reducing agents on characteristics and antimicrobial activity of copper nanoparticles. Mater. Lett..

[60] Din M.I., Arshad F., Rani A., Aihetasham A., Mukhtar M., Mehmood H. (2017). Single step green synthesis of stable copper oxide nanoparticles as efficient photo catalyst material. Biomed. Mater..

[61] Wu S.H., Chen D.H. (2004). Synthesis of high-concentration Cu nanoparticles in aqueous CTAB solutions. J. Colloid Interface Sci..

[62] Rajesh K.M., Ajitha B., Reddy Y.A.K., Suneetha Y., Reddy P.S. (2016). Synthesis of copper nanoparticles and role of pH on particle size control. Mater. Today Proc..

[63] Armendariz V., Herrera I., Jose-Yacaman M., Troiani H., Santiago P., Gardea-Torresdey J.L. (2004). Size controlled gold nanoparticle formation by *Avena sativa* biomass: Use of plants in nanobiotechnology. J. Nanopart. Res..

[64] Sebastiammal S., Shally V., Priyadharshini M., Jayam G. (2017). Structural and optical properties of Cerium oxide nanoparticles. Int. J. Eng. Trends Technol..

[65] Varshney R., Bhadauria S., Gaur M.S., Pasricha R. (2011). Copper nanoparticles synthesis from electroplating industry effluent. Nano Biomed. Eng..

[66] Cullity B.D. (1978). Elements of X-ray Diffraction.

